# The impacts of withdrawal and replacement of general practitioner services on aeromedical service trends: a 13-year interrupted time-series study in Tennant Creek, Northern Territory

**DOI:** 10.1186/s12913-015-1110-y

**Published:** 2015-10-05

**Authors:** Matthew T. Haren, John Setchell, David L. John, Mark Daniel

**Affiliations:** School of Population Health, Division of Health Sciences, University of South Australia, Adelaide, SA Australia; Spatial Epidemiology and Evaluation Research Group, Sansom Institute for Health Research, University of South Australia, Adelaide, SA Australia; The Royal Flying Doctor Service (RFDS) of Australia, Central Operations, Mile End, SA Australia

**Keywords:** Rural health, Remote health, Aeromedical services, Primary healthcare services, Accessibility, Interrupted time-series, Royal flying doctor service

## Abstract

**Background:**

The Royal Flying Doctor Service (RFDS) provides aeromedical care to patients during fixed-wing transport over vast distances to healthcare unavailable in rural or remote communities. This study examined the relationship between changes in local accessibility to primary healthcare services and rates of aeromedical service use over time.

**Methods:**

This was a 13-year interrupted time-series study (1999–2012) in Tennant Creek, Northern Territory. Quarterly aeromedical service use for primary care sensitive conditions were calculated and exposure to general practice (GP) services was characterised over time with events modelled as intervention variables: (a) GP service withdrawal (Nov-2004); and (b) GP service replacement (Dec-2006). Intervention effects were estimated using PROC ARIMA in SAS after examination of the time-series structure.

**Results:**

GP withdrawal resulted in an immediate and sustained doubling in quarterly aeromedical service use (+11.8 services per quarter) and GP service replacement had no significant effect.

**Discussion:**

Large and immediate increases in aeromedical service use result from the loss of local GPservices yet, in this case, replacement with a new GP service, 2-years hence, did not ameliorate that effect after six years.

**Conclusions:**

These findings demonstrate the immediate impact of GP-service loss on the rates ofaeromedical transfer of patients from this remote community and lend caution to expectations about thetimeline over which newly implemented primary health care services in such contexts can mitigate the impact of such a loss.

## Background

The alignment of accessibility to appropriate, effective and comprehensive primary healthcare services and local healthcare service need is a common issue for health systems globally and particularly pertinent to remote population settings where reduced economies of scale make service provision relatively inefficient. In Australia, current national health reform includes amongst its goals, the improvement of such alignment in rural and remote communities [1]. This can theoretically be achieved in two ways: (1) by intervening directly on local service availability and accessibility (the public health services focus); and (2) by intervening on community risk conditions to reduce the background health risk (the focus of health promotion) for which service delivery must cater.

In rural and remote Australia, a key service bridging the gap between locally accessible health services and service need are the fixed-wing aeromedical services provided by the Royal Flying Doctor Service (RFDS). Primary evacuation services transport sick or injured persons from locations with no or very low-level health service infrastructure to definitive (usually secondary or tertiary hospital) care. For individuals, a primary evacuation may be indicative of an unmet need for multidisciplinary assessment and better care planning or specialist shared care; at least in the context of frequent aeromedical service users [2]. Inter-hospital transfers (IHTs) originate from rural hospitals of varying capacities and transport patients to higher level (usually tertiary level) hospital care. For a community, IHT rates are associated with the size of the hospital at the location of flight origin in a condition-specific pattern [3]: IHTs for trauma decline whilst those for respiratory diseases increase marginally with increasing size of the originating hospital; IHTs for cardiovascular diseases are independent of hospital size. Beyond this, little else is known about the relation of local service accessibility to aeromedical service use.

The objective of this study was to examine the response in the aeromedical service trend for primary care sensitive conditions, to changes in the local accessibility to general practitioner (GP) services over 13 years in the non-indigenous resident population of Tennant Creek in Australia’s Northern Territory. This study capitalises on a natural experiment of withdrawal of a long-standing GP and the introduction (2-years later) of a new GP practice operated by the RFDS.

### Conceptual framework

This study conceptualises the gap between local health service accessibility and health service need, in rural and remote Australia as being largely reflected in the rate of aeromedical service use attributed at the local level. Health service need in this context is considered to be the background health risk resulting from the interaction between personal factors and environmental risk conditions attributable to rural and remote places in which people live [4, 5]. This facilitates recognition of social, physical and built environments (in addition to primary healthcare) as levers to close the gap between service accessibility and need in order to improve population health status. Accessibility, for the purpose of this study is operationalised in terms of local geographic availability, recognising that other services are available in the region (for example 510 km south in Alice Springs), but not as accessible as services provided within the geographical bounds of the community.

## Methods

### Research setting

Tennant Creek (population 3,500), is located on the Stuart Highway 510 kilometres (km) north of Alice Springs and 670 km south of Katherine. It services ‘The Barkly’ tableland, an area of similar size to the UK or New Zealand (240,000 km^2^) located between the tropical 'Top End' and the arid 'Red Centre' of Australia’s Northern Territory. It consists largely of open grass plains with cattle stations, mines and Indigenous communities.

Health service infrastructure in Tennant Creek includes a twenty-bed hospital which provides services to assess, diagnose and treat short-term illnesses and injuries, as well as an eight-chair renal dialysis unit. The primary healthcare needs of the Aboriginal community of Tennant Creek have long been served by the local Aboriginal Medical Service.

### Characteristics of the natural experiment

Accessibility to local primary healthcare for non-Aboriginal residents of Tennant Creek was, prior to November 2004, maintained continually by a solo male GP (1.0FTE) who ceased practicing at that time (Intervention A). Through to December 2006 the town lacked local access to a GP service, prompting the introduction of the Tennant Creek General Practice Service (TCGPS) (Intervention B) which was established by the RFDS (Central Operations) at the request of local authorities and supported by a Memorandum of Understanding between key Commonwealth, Territory and local stakeholders. Operating with a solo male GP (1.0FTE) the purpose of the TCGPS was to increase local access to comprehensive primary healthcare services to residents and visitors of Tennant Creek and surrounding Barkly region. Its services included medical examinations and health screening, preventative health, antenatal and counselling services as well as other RFDS-operated health and education programs such as the Rural Women’s General Practitioner Service. The TCGPS was primarily a full fee paying practice with concessions available to healthcare card holders (government health care assistance) and children under the age of 16. On average, since the second quarter of operation, the TCGPS consistently performed 930 patient consultations per quarter including 64 new patients per quarter. There was no data available to the study on the quantum of activity performed by the solos GP practice prior to November 2004.

### Outcome measurement

De-identified unit records were retrieved from the RFDS Central Operations Flight database. All records with flights originating in Tennant Creek (International Civil Aviation Organisation airport code YTNK) for the life of the database (1999q2 to 2012q3) were extracted. Extraction was restricted to the non-Aboriginal resident population of Tennant Creek (i.e. the underlying population on which the interventions were design to primarily impact). Consistent with the expected health benefits of primary healthcare [6, 7], outcomes were defined in terms of aeromedical service events for primary care sensitive conditions [8] adjudged at the ICD-9 chapter heading level. For example, it was not deemed that the service interventions would logically or theoretically have an impact on ‘Injuries and Poisoning’ (ICD-9 800–999) thus aeromedical services with these condition codes were excluded.

Despite the remarkably stablenon-Aboriginal population in Tennant Creek between 1999 and 2012, quarterly aeromedical service use was analysed both as raw counts and rates (calculated as a proportion of the non-Aboriginal Tennant Creek population as at the 2001, 2006 and 2011 Australian Census’ for records occurring between 1999 to 2003; 2004 to 2008; and 2009 to 2012 respectively). Further, despite the sex distribution remaining stable, the population had aged and to account for such population shifts over time and any effect these might have on propensity for aeromedical service use, quarterly rates were standardised to the age and sex distribution of the non-Aboriginal population of Tennant Creek in 2011 using direct standardisation. The study protocol was approved by the Human Research Ethics Committee of the University of South Australia.

### Analytical approach

#### Identification of the pre-intervention models

This study involves two interventions, thus two pre-intervention models were built sequentially:

- Pre-intervention A series from 1999q2 through 2004q3 (22 quarterly observations)
- Pre-intervention B series from 1999q2 through 2006q3, adjusted for Intervention A effect (30 quarterly observations) i.e. the Intervention A model

**Fig. 1:**
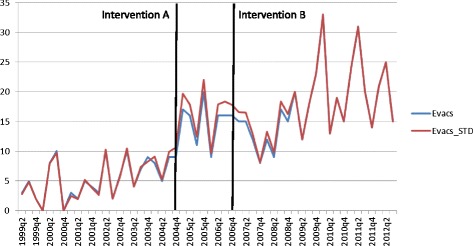
Quarterly RFDS aeromedical service occurrences of the non-aboriginal population from Tennant Creek excluding ‘Repatriation/Healthy’ and ‘Injury/Poisoning’, June 1999 to December 2012 (showing time of interventions). Evacs (blue series) is the raw aeromedical service count; Evacs_STD (red series) is the age and sex standardised aeromedical service count

The error structure (trend, autocorrelation, and seasonality) [9–11] was examined using Autoregressive Integrated Moving Average (ARIMA) models in SAS version 9.3 [12], which were used for all analyses unless otherwise stated. Figure 1 shows the time series of aeromedical services originating from Tennant Creek between 1999q2 and 2012q3. The pre-intervention A series showed a very slight upward trend, however the autocorrelation function (ACF) revealed no evidence of a slow decaying pattern indicating differencing was not necessary. The ACF showed a correlation at lag 3 (ρ = 0.33), as did the partial ACF indicating a simple MA(3) or ARIMA (0,0,3) model, however estimation of the MA(3) term showed it to be insignificant (*p* = 0.137) leaving a simple regression model with a mean term only:

$$ \mathrm{P}\mathrm{r}\mathrm{e}-\mathrm{intervention}\ \mathrm{A}\ \mathrm{model}:\ {\mathrm{Y}}_{\mathrm{t}}=\upmu + {\mathrm{a}}_{\mathrm{t}} $$

where: Y_t_ is the service rate at time t, μ is the mean around which the series fluctuates and a_t_ is the error term at time t.

Analysis of the residuals indicated they could be assumed as normal and the Ljung-Box statistics [13] indicated no significant residual autocorrelation. Final estimates for the pre-intervention A model were:

**Table:** 

	coefficient	*p*-value
Quarterly mean (μ)	5.196	<0.001
Correlation in residuals (Q_6_)	3.47	0.748
Correlation in residuals (Q_12_)	9.58	0.652

where: Q_6_ and Q_12_ are the residual Ljung-Box statistics.

### Intervention specification

Three intervention models of the form suggested by Box and Tiao [14] were considered based on the possible impacts Intervention A (GP service withdrawal) and B (GP service replacement) could have on the aeromedical service rate: (1) abrupt and permanent; (2) gradual and permanent; and (3) abrupt and temporary. To test these potential effects, exposure of the population to the intervention was specified (as dummy variables) as follows:

- For the ‘abrupt and permanent’ and ‘gradual and permanent’ shifts:
  0 before the intervention quarters, 0.33 in the quarter of intervention implementation (as both Interventions A and B occurred one-third of the way through a quarter), and 1 thereafter

(b) For the ‘abrupt and temporary’ shift:
  0 before the intervention quarters, 1 during the intervention quarter and 0 thereafter.

Thus, the following Intervention A models were tested:

Model 1: Abrupt and permanent impact: Y_t_ = μ + ω_1_I_1t_ + a_t_

Model 2: Gradual and permanent impact: Y_t_ = μ + [ω_1_/(1 − δΒ)] I_1t_ + a_t_

Model 3: Abrupt and temporary impact: Y_t_ = μ + [ω_1_/(1 − δΒ)] I_2t_ + a_t_

where: Y_t_ is the aeromedical service rate at time t,

I_1t_ is the intervention A dummy variable coded as (a) above

I_2t_ is the intervention A dummy variable coded as (b) above

ω_1_ is the effect of intervention A: i.e. the net difference in the level of the time series before and after the introduction of the intervention A (for abrupt and permanent impact); the change in the level of time series at the moment of intervention A (for the other two models)

δ is a rate parameter describing how quickly (or slowly) the effect is realised (in the case of the gradual and permanent impact) or decays to zero (in the case of the abrupt and temporary impact)

Β is the “backshift operator” such that, for example, BYt = Yt-1

Following this, the best Intervention A model (now considered the pre-intervention B model) was expanded to estimate the effects of Intervention B as follows (e.g. using Intervention A, Model 1: Y_t_ = μ + ω_1_ I_1t_ + a_t_).

Model 1: Abrupt and permanent impact: Y_t_ = μ + ω_1_I_1t_ + ω_2_I_2t_ + a_t_

Model 2: Gradual and permanent impact: Y_t_ = μ + ω_1_I_1t_ + [ω_2_/(1 − δΒ)] I_2t_ + a_t_

Model 3: Abrupt and temporary impact: Y_t_ = μ + ω_1_I_1t_ + [ω_2_/(1 − δΒ)] I_3t_ + a_t_

where: Y_t_ is the aeromedical service rate at time t,

I_1t_ is the intervention A dummy variable coded as (a) above

I_2t_ is the intervention B dummy variable coded as (a) above

I_3t_ is the intervention B dummy variable coded as (b) above

ω_1_ is the effect of intervention A: i.e. the net difference in the level of the time series before and after the introduction of the intervention A (for abrupt and permanent impact); the change in the level of time series at the moment of intervention A (for the other two models)

ω_2_ is the effect of intervention B: i.e. the net difference in the level of the time series before and after the introduction of the intervention B (for abrupt and permanent impact); the change in the level of time series at the moment of intervention B (for the other two models)

δ is a rate parameter describing how quickly (or slowly) the effect is realised (in the case of the gradual and permanent impact) or decays to zero (in the case of the abrupt and temporary impact)

Β is the “backshift operator” such that, for example, BYt = Yt-1

## Results

Between 1999q2 and 2012q3 a total of 675 aeromedical service occurrences originated from Tennant Creek for what were considered to be primary care sensitive conditions in the non-Aboriginal resident population. Figure 1 shows the 13-year trend in raw and standardised quarterly aeromedical service occurrences for these conditions combined.

Intervention analysis (Table 1) showed that Intervention A was associated with an abrupt and sustained (Model 1) average quarterly increase of close to 12 aeromedical service events over the following seven quarters (ω_1_ = 11.77, *p* < 0.001) and that Intervention B did not impact significantly on the time series (ω_2_ = 1.326, *p* = 0.510). These models were repeated with the aeromedical service rate expressed as a proportion of the overall rate across the RFDS Central Operations zone (SA and NT), to adjust for macro-level (but not Tennant Creek specific) events coinciding with the timing of the interventions. These yielded equivalent results to the models reported in Table 1.

**Table 1:** Intervention models of ‘abrupt and permanent’ impact on standardised quarterly aeromedical service counts from Tennant Creek, Northern Territory

Model 1: Abrupt and permanent impact				
	Intervention A model		+Intervention B model	
parameters	coefficient	*p*-value	coefficient	*p*-value
Quarterly mean (μ)	5.245	<0.001	5.245	<0.001
Intervention A effect (ω_1_)	11.710	<0.001	11.771	<0.001
Intervention B effect (ω_2_)			1.326	0.5104
Correlation in residuals (Q_6_)	4.42	0.6195	11.19	0.0827
Correlation in residuals (Q_12_)	10.01	0.6153	17.96	0.1168

Intervention A = GP withdrawal; Intervention B = TCGPS introduction

The two other intervention responses considered; gradual and permanent impacts (Model 2) and abrupt and temporary impacts (Model 3) were not supported. Despite a significant average quarterly increase of fourteen aeromedical services over the seven quarters following Intervention A (ω_1_ = 14.04, p = 0.017), Model 2 was rejected in favour of Model 1 due to an insignificant rate term (δ = −0.228, *p* = 0.646) suggesting that the effect did not occur gradually. Model 3 failed to result in stable parameter estimates despite various estimation methods and tolerance options and was not considered further.

The Intervention B period appeared to coincide with greater variability in the quarterly aeromedical service rates. To examine whether this might be related to variation in TCGPS activity the ‘Gradual and permanent impact’ model (Model 2) was also tested for Intervention B using the quarterly number of new TCGPS patients and total number of quarterly TCGPS consultations as intervention variables, but as with the simple intervention dummy variable, no stable parameter estimates were obtained.

All analyses were performed using both count and rate data which returned the same results.

Comparing the primary clinical reasons for aeromedical services between pre-intervention A, Intervention A and Intervention B periods (Table 2), most notably the proportion of ‘Genitourinary system’ conditions increased after Intervention A and returned to pre-intervention A levels after Intervention B. The proportion of ‘Mental disorders’ was lower after Intervention B when compared with pre-intervention A and Intervention A periods. Across the three time-periods, there appeared to be a declining trend in the proportion of ‘Circulatory system ‘conditions and increasing proportions of ‘Ill-defined conditions’ and ‘Respiratory system’ conditions.

**Table 2:** The contribution of primary care sensitive conditions to aeromedical services from Tennant Creek, by intervention period

	Pre-Intervention A (1999q2 - 2004q3)		Intervention A (2004q4 - 2006q3)		Intervention B (2006q4 - 2012q2)		Total	
ICD9 Description	n	%	n	%	n	%	n	%
Not stated	4	3.5	2	1.8	4	0.9	10	1.5
Circulatory system	33	28.9	29	25.4	100	22.4	162	24.0
Congenital abnormalities	0	0.0	0	0.0	1	0.2	1	0.1
Digestive system	14	12.3	13	11.4	71	15.9	98	14.5
Diseases of the Blood	1	0.9	0	0.0	4	0.9	5	0.7
Endocrine	3	2.6	1	0.9	3	0.7	7	1.0
Genitourinary system	2	1.8	9	7.9	11	2.5	22	3.3
Ill-defined conditions	13	11.4	17	14.9	87	19.5	117	17.3
Infections and Parasitic diseases	0	0.0	1	0.9	2	0.4	3	0.4
Mental disorders	12	10.5	11	9.6	24	5.4	47	7.0
Musculoskeletal system	1	0.9	2	1.8	12	2.7	15	2.2
Neoplasms	0	0.0	1	0.9	4	0.9	5	0.7
Nervous system	4	3.5	4	3.5	13	2.9	21	3.1
Perinatal	2	1.8	0	0.0	0	0.0	2	0.3
Pregnancy	14	12.3	7	6.1	38	8.5	59	8.7
Respiratory system	10	8.8	12	10.5	58	13.0	80	11.9
Skin and subcutaneous tissue	1	0.9	5	4.4	15	3.4	21	3.1
Total	114	100.0	114	100.0	447	100.0	675	100.0

Conditions are categorised by ICD9 chapter heading descriptions. Aeromedical service occurrences for this purpose exclude those coded as ‘Repatriation/Healthy’ and ‘Injury and Poisoning’

## Discussion

This study has demonstrated a significant abrupt and sustained increase of approximately 12 aeromedical services per quarter (greater than 100 % increase) from Tennant Creek associated with GP service withdrawal. This increase was not reversed in response to the introduction of the replacement GP service (the TCGPS). There is visual evidence of an increase in variability in the six years after this event which did not appear due to quarterly variations in the number of new TCGPS patients or total TCGPS consultations.

These findings suggest that local access to primary healthcare can have a large impact on a community’s reliance on aeromedical services however the relationship is complex and likely subject to contextual factors not able to be explored in this study. Whilst the increased reliance on aeromedical services due to the withdrawal of the only accessible GP service is expected, the absence of an observable opposing response to the introduction of a new GP service is important. Firstly, it potentially reflects impacts of service discontinuity and suggests that even a 2-year service gap can have a prolonged effect on the management of health risk at a community level. A systematic review of primary healthcare attributes related to patient satisfaction, better health outcomes and cost effectiveness demonstrated that continuity was a key attribute related to all three performance indicators [15].

In communities who have experienced discontinuity in local access to primary healthcare, the introduction of a new service would be expected to reduce the reliance on aeromedical services over time due to expectations that both the patient base of the service becomes stable (fewer new patients) and that chronic disease is better managed locally. ‘How long this takes and for what degree of population health gain?’ is the key health service policy question. The current study shows that in Tennant Creek, reliance on aeromedical services has not reduced after 6 years of the TCGPS. This is a mixed finding as it likely reflects patients being referred to appropriate care sooner than may have otherwise occurred in the absence of local access to primary healthcare. However, it may also reflect a partial shortcoming in the capacity of the service to manage some conditions and health risks locally. What is missing from this study, is knowledge of the health gain (or loss) associated with the aeromedical service rate. This will be the subject of future research. At the time this manuscript was being finalised and following a review of GP service provision in Tennant Creek it was decided that the TCGPS would be taken over by the NT Department of Health and re-located to the Tennant Creek hospital; it ceased operation on the 27th September 2013.

Primary healthcare represents a key component of a functioning health system, for which improvement in local accessibility has been demonstrated to reduce potentially avoidable hospitalisations in Canadian First Nations’ communities in Manitoba [16]. Although Lavoie’s work and ours differs in the focus on Indigenous and non-Indigenous populations respectively, it is relevant through the common issues of servicing remote communities locally. Beyond these two studies, published literature on the effect of improved access to primary healthcare in remote, underserved populations on intermediary indicators or health outcomes is sparse.

Like Lavoie and colleagues [16] we were unable to test for condition-specific responses to the interventions due to the low frequency of aeromedical service events when stratified by condition. Descriptively, the proportional contribution of conditions of the ‘Genitourinary system’ to total aeromedical transfers appeared to associate most strongly with periods of GP service access (1.8 % and 2.5 % during solo GP and TCGPS service respectively and 7.9 % during the 2-year service gap). The proportional contribution of transfers for ‘mental disorders’ appeared lower specifically during the TCGPS (intervention B) period, suggesting improved local management of this category of conditions.

With interrupted time-series designs, threats to internal validity [17] require consideration. Firstly, *History* – it is possible that events co-occurred with the intervention A which may account for the observed change in the aeromedical service trend. This study took account of any changes to the age and sex structure of the underlying population but was not able to account for potential changes to environmental risk conditions such as the introduction or loss of other services and facilities. One such explanation for the apparent increase in the variability of quarterly aeromedical service rates after the introduction of the TCGPS was instability in hospital staffing which could conceivably produce variation in IHT decisions. Analysis was repeated with the aeromedical service trend expressed as a proportion of the overall trend across the RFDS Central Operations zone (SA and NT), which served to adjust for macro-level (but not Tennant Creek specific) events coinciding with the timing of the intervention. This analysis yielded equivalent findings. Secondly, *Testing* –standard recording of aeromedical services is not likely to itself have an effect on the aeromedical service use of the Tennant Creek population. Thirdly, *Instrumentation* – the data base for storing aeromedical service data underwent migration and upgrade in 2004q2. An intervention term was modelled and found to be non-significant and was therefore removed from the models. *Instability –* there were constant changes to the local AMS and hospital accident and emergency services over the course of this study which may be partly related to the degree of variability in aeromedical service use observed across the time-series. The variability in the time-series appeared to increase around the time the TCGPS was introduced which may explain the inability of Intervention B models 2 and 3 to return stable estimates. This degree of variability (or instability) lends weight to the validity of the observed intervention A effect, however it could mean that a true Intervention B effect could have gone undetected.Lastly, *control of implementation of intervention* is unlikely to be a threat to the validity of the current findings as intervention dates and service use of the TCGPS were well characterised. Moreover, it is unlikely that any planning or organisational activity in the lead up to the introduction of the TCGPS would have affected the aeromedical service trend. S*election* is not a threat to the internal validity of this study, given the ‘full-capture’ nature of the aeromedical services data, however considering external validity, it is possible that the findings may be particular to this community context and that such changes to local accessibility to primary care in a different rural or remote Australian community may have a different effect on the aeromedical service trend.

## Conclusions

This is the first investigation into the response in aeromedical service rates to changes in local accessibility to primary healthcare (GP) services in rural and remote Australia. Findings suggest large and immediate increases in aeromedical service use result from the withdrawal of local GP services yet in this case, replacement with a new GP service, two years hence, did not ameliorate that effect after six years of service. These findings are immediately relevant to the current Australian national health reform which aims to improve the alignment of local access to comprehensive and effective primary health care with health service needs in rural and remote communities. These findings lend caution to expectations about the timeline over which newly implemented primary health care services in remote communities with poor service access can begin to impact on the need to for non-accident and injury related aeromedical service transfers.

## Abbreviations

RFDS: Royal flying doctor service
TCGPS: Tennant creek general practice service
SA: South Australia
NT: Northern Territory
GP: General practitioner
ICD: International classification of diseases
IHT: Inter-hospital transfer
ARIMA: Autoregressive integrated moving average
ACF: Autocorrelation function
RRHSF: Rural and remote health strategic framework
